# Probiotic bacteria can modulate immune responses to paratuberculosis vaccination

**DOI:** 10.3389/fcimb.2024.1394070

**Published:** 2024-06-04

**Authors:** Maddi Oyanguren, Elena Molina, Maitane Mugica, Iraia Ladero-Auñon, Miguel Fuertes, Miguel Fernández, Julio Benavides, Natalia Elguezabal

**Affiliations:** ^1^ Department of Animal Health, NEIKER-Basque Institute for Agricultural Research and Development- Basque Research and Technology Alliance (BRTA), Derio, Bizkaia, Spain; ^2^ Departamento de Sanidad Animal, Facultad de Veterinaria, Universidad de León, León, Spain; ^3^ Departamento de Sanidad Animal, Instituto de Ganadería de Montana (IGM) Consejo Superior de Investigaciones Científicas-Universidad de León (CSIC-ULE), León, Spain

**Keywords:** paratuberculosis, vaccine, trained immunity, probiotic, neutrophils, reactive oxygen species, phagocytes, macrophage polarization

## Abstract

*Mycobacterium avium* subsp. *paratuberculosis* (Map) is the etiological agent of paratuberculosis (PTB), a chronic intestinal inflammatory disease that causes high economical losses in dairy livestock worldwide. Due to the absence of widely available preventive or therapeutical treatments, new alternative therapies are needed. In this study, the effect of a probiotic alone or in combination with a commercial vaccine has been evaluated in a rabbit model. Vaccination enhanced the humoral response, exerted a training effect of peripheral polymorphonuclear neutrophils (PMNs) against homologous and heterologous stimuli, stimulated the release of pro-inflammatory cytokines by gut-associated lymphoid tissue (GALT) macrophages, and reduced the bacterial burden in GALT as well. However, the administration of the probiotic after vaccination did not affect the PMN activity, increased metabolic demand, and supressed pro-inflammatory cytokines, although humoral response and bacterial burden decrease in GALT was maintained similar to vaccination alone. The administration of the probiotic alone did not enhance the humoral response or PMN activity, and the bacterial burden in GALT was further increased compared to the only challenged group. In conclusion, the probiotic was able to modulate the immune response hampering the clearance of the infection and was also able to affect the response of innate immune cells after vaccination. This study shows that the administration of a probiotic can modulate the immune response pathways triggered by vaccination and/or infection and even exacerbate the outcome of the disease, bringing forward the importance of verifying treatment combinations in the context of each particular infectious agent.

## Introduction

Paratuberculosis (PTB), which is caused by *Mycobacterium avium* subsp. *paratuberculosis* (Map), is a chronic granulomatous enteritis that affects domestic ruminants worldwide, causing economic losses in livestock and poor animal wellness (Rasmussen et al., 2021). It has been associated with a variety of human diseases, but especially with Crohn’s disease (Juste et al., 2008) due to similarities in disease presentation, although this relation is still controversial. In any case, detection of Map DNA in dairy products (Sevilla et al., 2017) should increase the awareness of this disease and its importance in public health.

Map is an intracellular pathogen that is mainly transmitted through the fecal–oral route. Neonates and young animals are most susceptible to Map, getting infected in the early stages of life, developing granulomatous lesions in the gastrointestinal tract, and manifesting clinical signs such as diarrhoea, emaciation, and production decrease approximately 2 years after infection (Arsenault et al., 2014).

Currently, vaccination is the best and most economic strategy for PTB control. Inactivated vaccines have been shown to be useful in both sheep (Bastida and Juste, 2011; Links et al., 2021) and cattle (Juste et al., 2021). Vaccination is effective in controlling the infection by reducing the severity of lesions, bacterial load, elimination of Map through feces (Juste et al., 2009), reducing the mortality of heifers by 35%, and extending productive life when vaccination takes place in their first 3 months of life (Juste et al., 2021). This non-specific effect on total mortality associated with PTB vaccination in cattle has been proposed to be due to the trained immunity of innate cells. Furthermore, this heat-inactivated commercial vaccine has shown heterologous protection against other mycobacteria in experimental conditions (Serrano et al., 2017b; Ladero-Auñon et al., 2021). Despite all these benefits, the disease does not reach complete eradication even after years of vaccination (Bastida and Juste, 2011), and this vaccine is authorized only in a few countries due to the interference with bovine tuberculosis (bTB) diagnosis in eradication programs (Serrano et al., 2017a).

Probiotics have been proposed as supplements that can increase production and improve overall health in cattle (Uyeno et al., 2015). The FAO/WHO (2002) defines probiotics as “live microorganisms that confer a health benefit on the host when administered in adequate amounts”. Probiotics are live non-pathogenic organisms that can benefit host health by competition with other pathogenic microbes (Uyeno et al., 2015). Additionally, probiotics can improve mucosal immunity through the activation and stimulation of immune cells (Chaucheyras-Durand et al., 2012). The interest in probiotic research in ruminants has increased as these are seen as an alternative to traditional antibiotic use. Furthermore, probiotic administration has been proposed to increase vaccine efficacy (Kazemifard et al., 2022). Regarding PTB, a few studies have been conducted in this area (Click and Van Kampen, 2010; Badiei et al., 2013; Cooney et al., 2014). In field studies performed on PTB-positive cattle, *Dietzia* was used as a probiotic, showing ELISA value and clinical sign reduction after a daily dosage (Click and Van Kampen, 2010). In addition, *Dietzia* has been evaluated as a preventive therapy against PTB in newborn calves, showing promising results when Map challenge was performed in parallel to treatment (Click, 2011a). It would be interesting to know if prolonged *Dietzia* administration prior to Map challenge or encountering is effective as well. Moreover, considering that vaccination with Silirum^®^ does not confer absolute protection, it is worth exploring if *Dietzia* administered in combination with this vaccine can improve the protective activity of the vaccine since other studies developed in livestock in the context of intestinal pathogens have shown better results when combining vaccination and probiotic administration (Groves et al., 2021).

In an attempt to evaluate the possible preventive and synergistic or additive effect of *Dietzia*, treatments with *Dietzia* alone, in combination with vaccination with a commercial vaccine (Silirum^®^), and vaccination alone were evaluated in a well-established rabbit Map infection model to assess immunomodulatory and protective parameters.

## Materials and methods

### Ethics statement

All animal procedures were carried out following European and national and regional regulations on animals used for experimentation and other scientific purposes. The protocols were evaluated and approved by the Ethics Committee at NEIKER (NEIKER-OEBA-2018–0001) and authorized by the Regional Council (BFA-38012).

### Probiotic and bacteria inoculum preparation

All bacteria used for *ex vivo* and *in vivo* challenge assays were grown to the exponential phase at 37°C under aerobic conditions.

#### Preparation of Dietzia

*Dietzia* subsp. 79793–74 isolated from PTB-seropositive cattle feces with demonstrated *in vitro* Map growth-inhibiting ability (Richards et al., 1988) was kindly supplied by Dr. Robert Click. *Dietzia* was cultured for 48 h at 37°C, until the beginning of the exponential phase, in Trypticase soy broth (TSB) supplemented with fructose (Click and Van Kampen, 2010).

A batch of *Dietzia* was prepared to guarantee homogeneity and easy administration. Moreover, 10% of sucrose in PBS was added to the culture as cryopreservant. The culture was divided in glass vials in single dosages of 1 × 10^7^ colony-forming units (CFU), covered with paraffin, and stored at -80°C. Afterward, the vials were lyophilized for 24 h and kept at room temperature (RT) thereafter until administration. Aliquots of lyophilized *Dietzia* were resuspended in PBS and cultured in TSB to verify the viability, resulting in 70%–90% at 3 weeks after batch preparation.

#### Map for oral challenge

Cattle field strain Map 832 with demonstrated virulence in experimental conditions used for challenge was grown on Middlebrook 7H9 (7H9) liquid media supplemented with oleic albumin dextrose catalase (OADC) and mycobactin J (MJ) for 3 weeks at 37°C and adjusted to 5 × 10^8^ CFU/mL based on data from growth curves and plating considering that the 0.7 optical density (OD) is 1 × 10^8^ bacteria/mL for Map (Ladero-Auñon et al., 2021).

#### *Ex vivo* assays

*Staphyloccocus aureus* field strain was grown on brain heart infusion (BHI) broth for 24 h. The inoculum were adjusted after measuring the optical density at 600 nm and based on data from growth curves and plating considering that 0.4 OD is 2 × 10^8^ for *S. aureus* (Ladero-Auñon et al., 2021).

### Experimental design

**Figure 1:**
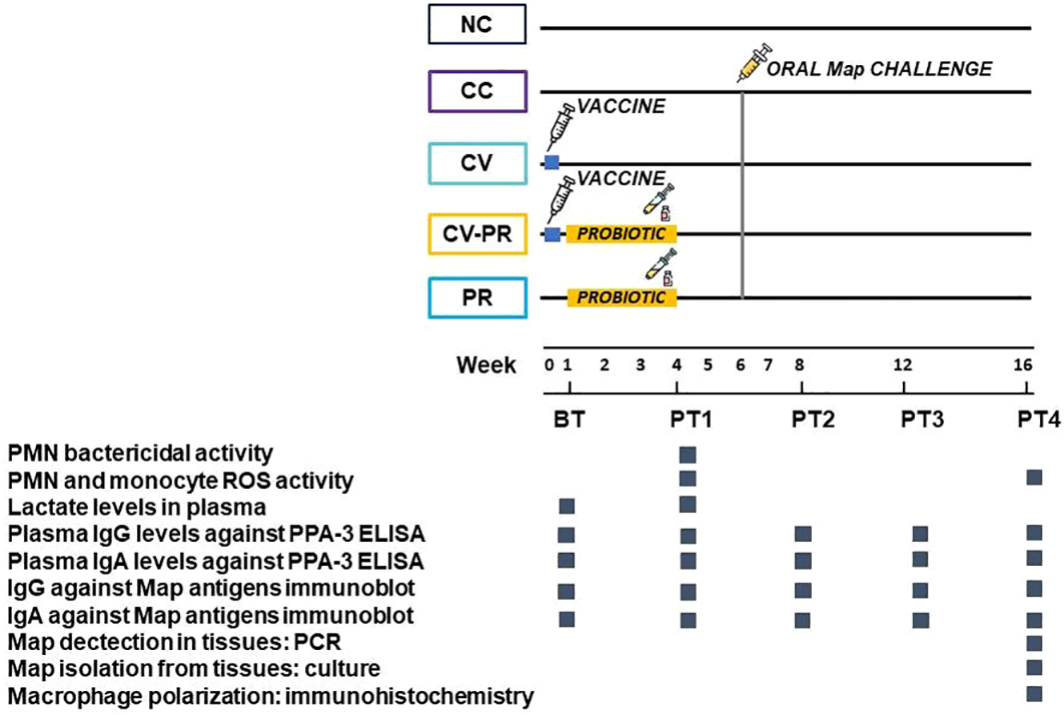
Experimental scheme showing interventions and analytics performed to monitor bacteriological and immunological parameters. The animals were divided in groups of *n* = 5: non-challenged control (NC), challenged control (CC), commercial vaccine (Silirum^®^) (CV), commercial vaccine + probiotic (CV-PR), and probiotic (PR) and sampled for different techniques before treatment (BT) and at different time points post-treatment (PT1–PT4).

A schematic representation of the experimental design is shown in **Figure 1**. New Zealand White female rabbits at 7 weeks of age were purchased from authorized experimental animal dealers (Granja Cunícola San Bernardo, Tulebras, Spain) and left on a 15-day acclimatization period. A total of 25 rabbits were divided into five groups of five animals each: non-vaccinated and non-challenged group (NC), challenged group (CC), subcutaneously vaccinated group with the commercially available vaccine Silirum^®^ (CV), a combined treatment group consisting of vaccination with the commercially available vaccine Silirum^®^ and probiotic administration (CV-PR), and finally an only-probiotic administration group (PR). The inactivated commercial vaccine was administered subcutaneously as a single dose (12.5 mg of antigen in 1 mL) on day 1. The probiotic was administered orally 3 days/week on alternate days during a total period of 4 weeks at a dose of 1 × 10^7^ CFU/day. Map oral challenge was performed on groups CC, CV, CV-PR, and PR on days 43, 44, and 45 by administering 1 × 10^9^ CFU/day. Blood sampling was performed monthly throughout the study to carry out different techniques depending on the sampling point: PMN isolation for bactericidal activity evaluation, reactive oxygen species (ROS) activity of PMNs and monocytes after stimulation with Map antigens, lactate quantification in plasma, IgG and IgA levels in plasma to Map protoplasmic antigen (PPA-3), and IgG and IgA levels in plasma to Map antigens. At the endpoint, 112 days (16 weeks) after the start of the experiment, all animals were euthanized by intracardiac administration of pentobarbital (200 mg/kg) (Vetoquinol^®^) after deep sedation with xylazine (5 mg/kg) (Calier^®^) and ketamine (35 mg/kg) (Merial^®^). Tissue samples from the sacculus rotundus (SR), vermiform appendix (VA), mesenteric lymph nodes (LN), ileum (IL), liver (L), and spleen (S) were then collected and stored at −20°C for Map isolation. Moreover, samples from SR were fixed in neutral-buffered formalin for immunohistochemical analysis.

### Peripheral polymorphonuclear neutrophil isolation

Peripheral polymorphonuclear neutrophils (PMNs) were isolated from 20 mL of blood passed through Histopaque^®^ 1119 as described previously (Ladero-Auñon et al., 2021). After the peripheral blood mononuclear cell (PBMC) layer was aspirated, and the entire volume of Histopaque^®^ 1119 was eliminated. PMNs localized in the pellet with remaining RBCs were aspirated to a new tube where hypotonic lysis was performed, and the samples were centrifuged at 300 g for 10 min. PMNs were suspended in rabbit leukocyte buffer and counted on a Bio-Rad TC20™ counter. This isolation protocol yields PMNs with >95% purity and >95% viability (Ladero-Auñon et al., 2021).

### Map sonicate preparation

Cattle field Map 764 was sonicated and used for *in vitro* stimulation as described previously (Ladero-Auñon et al., 2021). Briefly, Map was grown in 7H9 OADC MJ broth to exponential phase at 37°C for 3 weeks. The culture was centrifugated at 10,000 *g* for 20 min, and after two washes with cold PBS, the pellet was suspended in PBS and sonicated for three cycles of 5 min at 18 W on ice. Protein concentration was determined on a spectrophotometer and was adjusted to 100 µg/mL.

### Whole-blood reactive oxygen species production by phagocytes

Oxidative burst of PMNs and monocytes was evaluated using whole blood using the Respiratory Burst Assay kit (Abcam, ab236210) following the manufacturer´s instructions. Briefly, whole blood extracted with EDTA was mixed with 123 dihydrorhodamine (DHR) and incubated at 37°C in water bath for 15 min. Blood was mixed with PBS (non-stimulation), phorbol 12-myristate 13-acetate (PMA) (positive stimulus) and Map sonicate (specific stimulus), and incubated for 45 min at 37°C in a water bath. Afterward, RBC lysis was performed by adding the kit lysis buffer and incubating at 37°C in a water bath for 10 min. The samples were centrifuged at 500 g for 10 min at RT. The supernatant was aspirated, and the pellets were suspended in an assay buffer. Cell suspensions were analyzed using a CytoFLEX Flow Cytometer (Beckman Coulter). PMNs and monocytes were identified according to their specific FSC/SSC patterns. PMNs and monocytes producing reactive oxygen species (ROS) and converting DHR into rhodamine 123 were identified based on their fluorescence in the FITC channel. Data from the experiments were depicted as percentages of FITC-positive PMNs and monocytes of at least 10,000 events. Flow cytometry data were analyzed with CytExpert v2.3 software (Beckman Coulter). ROS percentages were expressed as the number of FITC-positive PMNs and monocytes divided by the total number PMNs and monocytes, respectively.

### PMN bactericidal activity

Isolated PMNs were seeded in tubes at 10^7^cells/mL in a total volume of 150 μL of RPMI 1640 (1×) with 15 μL of autologous serum and incubated for 15 min at 37°C. *S. aureus* was seeded at a multiplicity of infection (MOI) of 1 in a total volume of 165 μL. Immediately, 50 μL of volume (bacteria and PMNs) was removed to a new tube with 950 μL of cold PBS to stop the reaction and time zero was seeded. The rest was incubated in agitation for 30 min at 37°C. Afterward, tubes were put on ice to stop the reaction, and the samples were centrifuged at 150 g at 4°C for 10 min. The supernatant was removed and transferred to a new tube. The pellet was washed with 300 μL of cold PBS and centrifuged at 150 g for 10 min at 4°C. The obtained second supernatant was added to the first one, and 400 μL of PBS was added. The pellet was lysed with 150 μL Triton 0.1% disaggregated and incubated for 10 min at RT, and afterwards 850 μL of PBS was added to the lysed pellet. Finally, the extracellular bacteria from the supernatant and the intracellular bacteria from the lysed pellet were seeded. *S. aureus* CFUs were counted and recorded 18 h after seeding. The killing rate was calculated as 100 – [(extracellular bacteria CFU + intracellular bacteria CFU)/CFU grown in time zero) * 100].

### Lactate quantification assay

Lactate levels were analyzed in plasma samples taken before treatment (BT) and at 1 month after treatment (PT1). Lactate levels were quantified using the Lactate-GloTM assay (Promega) following the manufacturer’s instructions. Briefly, 50 μL plasma was diluted 1:100, and 10 twofold serial dilutions of a lactate standard were run in duplicate in 96-well plates. The assay was performed with the addition of 50 μL lactate detection reagent (0.25 μL reductase substrate, 0.25 μL reductase, 0.25 μL lactate dehydrogenase, and 0.25 μL NADH) and 50 μL luciferin detection solution. After incubation in the dark for 1 h at RT, luminescence was recorded using a plate-reading luminometer (Promega). The amount of lactate was determined by extrapolating the sample luminescence values from the standard curve, and the increase in lactate concentration (PT1-BT) was analyzed.

### ELISA PPA-3

Homemade indirect ELISA was performed using Map protoplasmatic antigen 3 (PPA-3) (Allied Monitor) as previously described (Arrazuria et al., 2016). Anti-rabbit IgA peroxidase (Abcam) and Protein G peroxidase (Sigma-Aldrich) were used as secondary reagents. Absorbance was measured at 405 and 450 nm using an automated ELISA plate reader (Multiskan EX^®^). The reading obtained at 450 nm was subtracted from the reading at 405 nm to reduce optical imperfections in the plate. The results were expressed as a relative absorbance index (quotient obtained of the division of the mean absorbance of the sample by the mean absorbance of the negative control sample).

### Map antigen detection by immunoblot

Map strain 832 was grown for 4 weeks in 7H9 OADC MJ medium at 37°C. At 8 OD, culture was washed with PBS 0.1 M, and full extraction buffer was added [buffer extraction, IP25 (protease inhibitor 25×), DTT (dithiothreitol)] and was placed in the Tissuelyser (Qiagen) with glass beads for Map protein extraction. Furthermore, 60 μg of protein extract was transferred to a polyvinylidene fluoride (PVDF) microporous membrane Immobilon^®^ (Millipore Ibérica, Madrid, Spain) by Trans-Blot^®^ Semi-Dry electrophoretic transfer cell (Bio-Rad Laboratories, Spain) at 15 V (Arrazuria et al., 2016). The PVDF membrane was cut in 0.5-cm large strips that were blocked overnight at 4°C with 0.1 M Tris-buffered saline (TBS) supplemented with 5% non-fat dry milk (TBS-M). Plasmas were diluted 1:50 in TBS-M before incubation with the membrane for 1 h at 37°C in slow agitation. After four 5-min washes with TBS, the membranes were incubated with recombinant protein G peroxidase (Sigma-Aldrich) 1:8,000 and Goat Anti-Rabbit IgA alpha chain peroxidase 1:5,000 (Abcam) in TBS-M for 1 h at 37°C. The membrane was washed four times more with TBS, and chemiluminescence reagent (ECL) was added. The immunoreactive bands were visualized by autoradiography, and after scanning the autoradiography, reactivity intensity was measured with Image J software (Arrazuria et al., 2016).

### Map isolation from tissues

For Map isolation, 0.5 g of tissues was collected and cultured on Herrold’s Egg Yolk Medium (HEYM) supplemented with MJ as described by Arrazuria et al. (2015). Mesenteric lymph node was spliced in tiny pieces and weighed, whereas VA, SR, L, SPL, and IL were scraped for the collection of mucosae and weighed, and the samples were treated as described previously (Arrazuria et al., 2015). Briefly, hexadecylpyridinium chloride (Sigma-Aldrich) 0.76% decontaminated suspensions were centrifuged at 2,885 *g* for 10 min. The supernatant was discarded, and the pellet was washed once with sterile water. After a last centrifugation step in the same conditions, the pellet was resuspended in 2 mL of water, and four drops/tube were seeded. All seeded tubes were incubated at 37°C. Map growth was examined at 8, 12, and 16 weeks.

Isolated colonies were confirmed by a real-time multiplex PCR detecting *IS900* and *ISMap02* Map sequences (Sevilla et al., 2014).

### Macrophage analysis by immunohistochemistry

Samples of SR were used for immunohistochemical analysis to characterize macrophage subpopulations and proliferation. For this purpose, different monoclonal antibodies against macrophage-expressed proteins were used (**Table 1**) to identify M1 and M2 subpopulations and macrophage proliferation. The SR samples fixed in formalin were conventionally processed for histological examination. Deparaffination and antigen retrieval were performed at 96°C with citrate buffer pH 6 or pH 9 solutions using a PT Link system (Dako, Agilent Technologies, Glostrup, Denmark). Afterward, endogenous peroxidase was inactivated by incubating in 3% hydrogen peroxide in methanol for 30 min. Subsequently, the slides were incubated with different primary antibodies overnight at 4°C in a dark and humidified chamber. After washing in PBS, the slides were incubated with EnVision + Dual Link System-HRP (Dako, Agilent Technologies, Santa Clara, CA, USA). After washing with PBS, antibody localization was determined using 3,3-diaminobenzidine (Sigma-Aldrich Corp., Madrid, Spain). Sections were counterstained with hematoxylin briefly and dehydrated through graded alcohol series. Slides were mounted with DPX (dibutyl phthalate xylene) and observed under a light microscope.

**Table 1:** Immune markers detected in sacculus rotundus by immunohistochemistry.

Antigen (clone)	Host	Specificity and target cells	Dilution	
***CD163 (anti-human)***	**Mouse**	**Expressed in M2 macrophages**		1:300
*MAC387* ***(anti-human)***	**Mouse**	Expressed in activated and recently recruited macrophages		1:200
*IFNγ* ***(anti-bovine)***	**Mouse**	Induces M1 macrophage polarization		1:100
*TNFα* ***(anti-bovine)***	**Mouse**	Expressed in M1 macrophages		1:250

Only immunolabeled macrophages present inside granulomas were considered and evaluated. For analysis, 20 representative fields through the whole slide containing granulomatous lesions were selected, photographed, and analyzed. The granulomas’ whole area and immunolabeled area were measured using Qupath software. For group comparison, the labeled areas were scored using an immunohistochemistry score (H-score) that incorporates both the staining intensity (i) and the percentage of stained cells at each intensity level (Pi), where i values range from 0 to 3 (0, none; 1, weak; 2, moderate; and 3, intense) and Pi values range from 0% to 100%. The H-score is ultimately calculated by summing up i multiplied by Pi as follows: (0xP_0_) + (1xP_1_) + (2xP_2_) + (3xP_3_). The H-score falls within the range of 0 to 300. The H-score was calculated by summing all areas of each intensity and multiplying by 1, 2, or 3 depending on the intensity as described before and dividing by the sum of the whole area of granulomas.

### Statistical analysis

Significance of the differences among groups for all variables: PMN and monocyte producing ROS percentage, PMN killing activity, lactate level increase (the difference between BT and PT1), ELISA OD levels, Map tissue PCR C*_t_* s, Map CFUs in tissues, and H-score were assessed using analysis of variance (ANOVA). When ANOVA showed significant differences, Tukey’s *post hoc* testing was used to make paired comparisons.

All statistical analyses were performed using Graph Pad Prism 9 statistical software (9.5.1), and differences among groups for all variables were stated at *p* < 0.05.

## Results

### ROS production by PMNs increases after 1 month of vaccination

**Figure 2:**
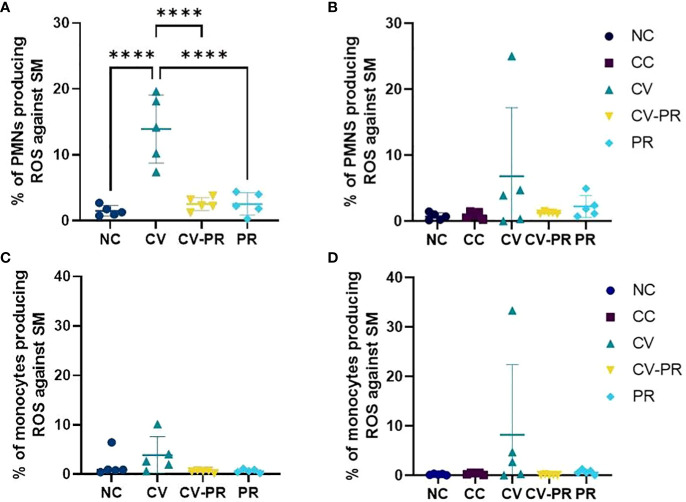
Reactive oxygen species (ROS) production of phagocytes. Percentage of ROS-producing phagocytes stimulated with Map sonicate (SM) in experimental groups: non-challenged controls (NC), challenged controls (CC), commercial vaccine (CV), commercial vaccine and probiotic (CV-PR), and probiotic (PR). **(A)** Neutrophil (PMN) ROS activity at PT1 (1 month post-treatment initiation), **(B)** PMN ROS activity at PT4 (4 months post-treatment initiation and 3 months post-challenge with Map), and **(C)** monocyte ROS activity at PT1 (1 month post-treatment initiation), **(D)** monocyte ROS activity at PT4 (4 months post-treatment initiation and 3 months post-challenge with Map). ANOVA with Tukey’s *post-hoc* test was applied, and the significant level was ****p < 0.0001.

For the evaluation of the training of the innate response, ROS production of phagocytes after stimulation with Map antigens was evaluated in whole blood. The ROS production results at PT1 (1 month after treatment) and PT4 (3 months after treatment) are shown on **Figure 2**. An increase of ROS production by PMNs and monocytes was observed only in the CV group against Map sonicate at 30 days post-treatment (PT1), although significant differences were only detected in the PMNs (*p* < 0.0001). This effect was maintained as a tendency at 50 days post-treatment PT2 (data not shown) and at 105 days post-treatment (PT4).

### PMNs from CV group show higher bactericidal capacity

**Figure 3:**
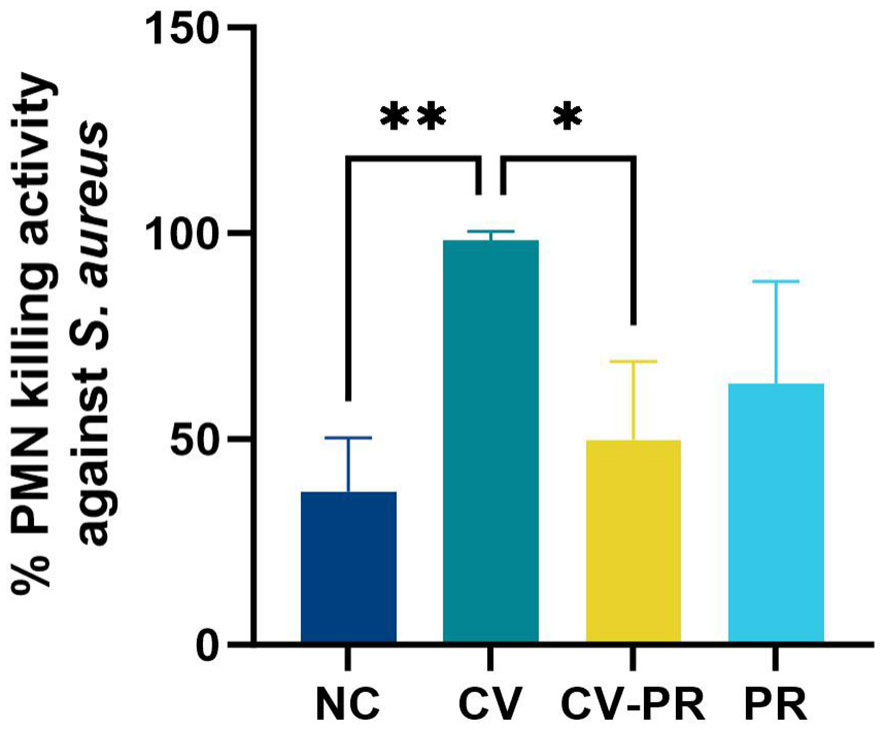
PMN bactericidal activity. PMN bactericidal activity against *Staphylococcus aureus* at PT1 (1 month post- treatment initiation) in experimental groups (*n* = 3) non-challenged controls (NC), commercial vaccine (CV), commercial vaccine and probiotic (CV-PR), and probiotic (PR). ANOVA with Tukey’s *post-hoc* test was applied, and the significant levels were **p < 0.01 and *p < 0.05.

Heterologous protection against another pathogen that affects ruminant livestock was evaluated by measuring the bactericidal capacity of PMNs against *S. aureus*. The killing rate of PMNs at PT1 (1 month after treatment initiation) is shown on **Figure 3**. The CV group showed the highest killing rate with significant differences compared to NC (*p* < 0.01) and CV-PR (*p* < 0.05).

### Vaccination in combination with the probiotic increases metabolic activity

**Figure 4:**
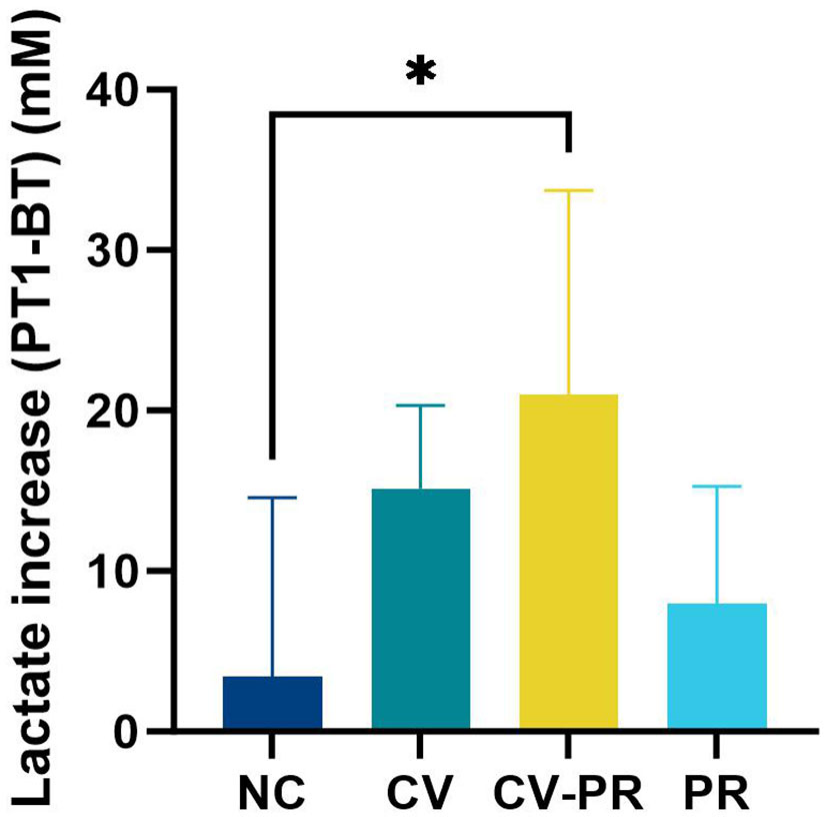
Lactate levels in plasma. Increase in lactate levels between PT1 (1 month post-treatment initiation) and BT (before treatment) in experimental groups: non-challenged controls (NC), commercial vaccine (CV), commercial vaccine and probiotic (CV-PR), and probiotic (PR). ANOVA with Tukey’s *post-hoc* test was applied, and the significant level was *p < 0.05.

In order to evaluate the immune activation produced by the different treatments, the lactate levels at BT (before treatment) and PT1 (1 month after treatment initiation) were measured (**Figure 4**). The lactate levels in plasma were increased by vaccination, although significant differences were only detected in the CV-PR group compared to the NC group (*p* < 0.05).

### Vaccination increases antibody levels against Map antigens

**Figure 5:**
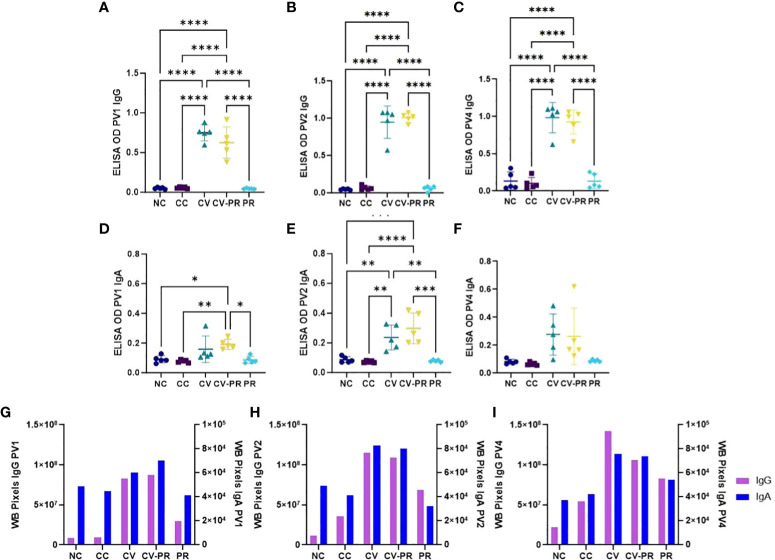
Humoral response to Map antigens. Antibody reactivity detection in experimental groups: non-challenged controls (NC), challenged controls (CC), commercial vaccine (CV), commercial vaccine and probiotic (CV-PR), and probiotic (PR). ELISA OD showing the mean values of *n* = 5 for each timepoint and standard deviation of anti-IgG reactivity to PPA-3 at **(A)** PV1, **(B)** PV2, and **(C)** PV4 and anti-IgA reactivity to PPA-3 **(D)**, PV1 **(E)**, and PV2 **(F)** PV4. ANOVA with Tukey’s *post-hoc* test was applied, and the significant levels were *p < 0.05, **p < 0.001, ***p < 0.0005, and ****p < 0.0001. Reactivity of immunoblot strips calculated with Image J software showing one value per experimental group and isotype (IgG and IgA) at **(G)** PV1, **(H)** PV2, and **(I)** PV4.

Humoral immune response was assessed by PPA-3 ELISA and by immunoblot against Map 832 strain extract. The anti-IgG and anti-IgA reactivities against PPA-3 are presented in **Figures 5A–F**, respectively. It was observed that both vaccinated groups, CV and CV-PR, presented a significant increase of both IgG (**Figures 5A–C**) and IgA (**Figures 5D–F**) antibody levels, in contrast with the NC, CC, and PR groups that did not show reactivity against PPA-3. Both CV and CV-PR animals presented similar levels throughout the experimental time points, suggesting that the administration of the probiotic and/or the challenge with Map did not interfere in the humoral response generated by vaccination.

To further analyze the humoral response, anti-IgG and anti-IgA against Map antigens extracted from strain 832 were evaluated by immunoblot (**Supplementary Figure S1**), and reactivity intensity was measured with Image J software and plotted (**Figures 5G–I**). Vaccination stimulated both IgG and IgA production in both vaccinated groups (CV and CV-PR), and the antibody levels increased throughout the experiment. The CV group showed the highest reactivity against Map antigens, followed by the CV-PR group. In the case of the CC and PR groups, the anti-IgG reactivity was increased after challenge and thereafter. It is worth highlighting that the PR group showed stronger anti-IgG and anti-IgA reactivity compared to the CC group at time PV4, indicating that the administration of the probiotic prior to challenge increased the antibody levels against Map.

### Bacterial burden in gut-associated lymphoid tissue is higher in the group treated only with probiotic

**Figure 6:**
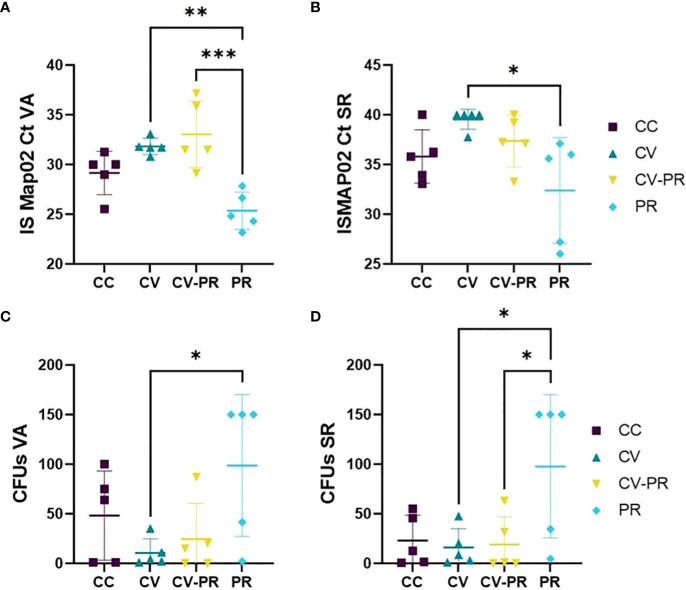
Map burden in gut-associated lymphoid tissue. Map detection or isolation in the experimental groups: challenged controls (CC), commercial vaccine (CV), commercial vaccine and probiotic (CV-PR), and probiotic (PR) by **(A)** Cts for PCR ISMAP02 in vermiform appendix (VA), **(B)** Cts for PCR ISMAP02 in sacculus rotundus (SR), **(C)** culture results expressed in colony-forming units (CFUs) in VA, and **(D)** culture results expressed in CFUs in SR. ANOVA with Tukey’s *post-hoc* test was applied, and the significant levels were *p < 0.05, **p < 0.001, and **p < 0.0001.

A lower Map presence detected in tissues by PCR and/or isolation in agar was used as an indicator of protection of the treatments. The tissues that contained high levels of Map were VA (**Figures 6A, C**) and SR (**Figures 6B, D**), although Map was also detected in SPL, LN, IL, and L (data not shown). The group treated with the probiotic (PR) presented the highest bacterial burden for both techniques. The highest amount of Map DNA (lowest C*_t_*) was detected in this group in both VA (**Figure 6A**) and in SR (**Figure 6B**). The same behavior was observed in VA (**Figure 6C**) and SR culture (**Figure 6D**), showing the highest CFUs. The CC group presented higher CFU levels in VA (**Figure 6C**) and similar CFUs as CV and CV-PR in SR (**Figure 6D**). Significant differences were observed in C*_t_* s in VA between CV and PR (*p* < 0.001) and between CV-PR and PR (*p* < 0.0001) and in SR between CV and PR (*p* < 0.05). Significant differences were observed in tissue culture, in VA (*p* < 0.05) and in SR (*p* < 0.05) between CV and PR. These results indicate that the probiotic alone administered in the conditions of the present study can be detrimental for Map elimination, even exacerbating bacterial burden. However, when administered in combination with the vaccine, it does not fully interfere with the protective effect as seen by similar bacterial burden levels observed in the CV-PR group compared to CV.

### Vaccination interferes with macrophage recruitment to the infection site

**Figure 7:**
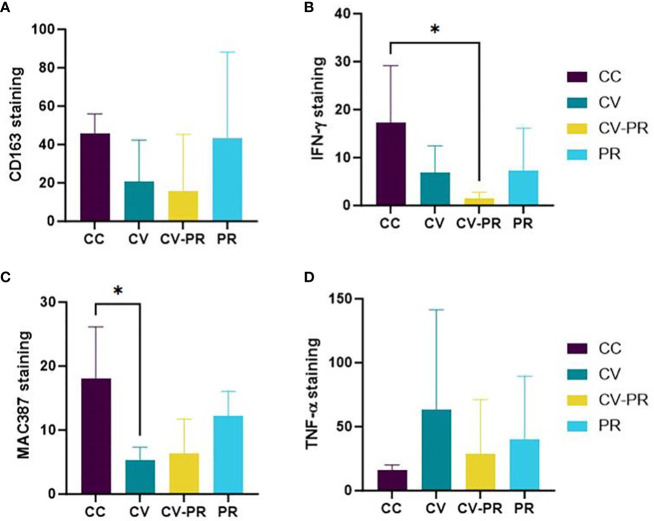
Macrophage polarization status. Mean immunohistochemistry score (H-score) in sacculus rotundus in the challenged controls (CC), commercial vaccine (CV), commercial vaccine and probiotic (CV-PR), and probiotic (PR) animals for **(A)** CD163, **(B)** IFNγ, **(C)** MAC 387- calprotectin, and **(D)** TNF-α. ANOVA with Tukey’s *post-hoc* test was applied, and the significant level was *p < 0.05.

Macrophage polarization and proliferation were analyzed for CD163, IFNγ, calprotectin, and TNF-α in the granulomatous lesions of SR by immunohistochemistry (**Supplementary Figure S2**). H-scores representing the intensity and percentage of immunolabeled areas for these macrophage markers in SR are presented in **Figure 7**.

The high individual variability has impeded the observation of differences between experimental groups. The only clearly observed differences have been the decrease in IFNγ in the CV-PR group (*p* < 0.05) (**Figure 7B**) and the decrease in calprotectin in the CV group (*p* < 0.05) (**Figure 7C**). The immunolabeling with the rest of the markers suggests that both vaccination and probiotic administration alter macrophage profiles, but not significantly in the settings of this experiment, 3 months after challenge. The H-score for CD163 was higher in the PR and CC groups, although significant differences were not observed between groups (**Figure 7A**). The H-score of TNF-α (**Figure 7D**) was highest in the CV group, although significant differences were not found between groups.

## Discussion

PTB vaccines have demonstrated beneficial effects in controlling the disease, and even beneficial non-specific effects have been reported, such as the reduction in the general mortality of cattle, suggesting an improvement in the herd health status (Juste et al., 2021). However, PTB vaccines do not provide total protection (Bastida and Juste, 2011), and their use interferes with bTB diagnosis. Therefore, other curative or preventive treatments are needed. The beneficial properties of probiotics for intestinal diseases have been studied in the past years. In the context of PTB, Protexin (a multistrain probiotic) lowered the relative risk of being infected by Map to four times less than calves that did not consume this probiotic during the first 90 days of life (Badiei et al., 2013), and *Lactobacillus casei* has shown promising results in mice (Cooney et al., 2014). Finally, *Dietzia* sp. 79793–74 administered at daily dosages in PTB-positive cattle (Click and Van Kampen, 2010; Click, 2011a, b) has been suggested as a curative treatment. In the study presented here, we attempted to evaluate if *Dietzia* also has beneficial effects when it is administered to animals prior to their contact with Map, as a preventive treatment, and if it has a synergistic or additive action in combination with a commercial vaccine (Silirum^®^) to see if it increases the vaccine efficacy. These treatments have been tested in a Map challenge rabbit model for their protective and immunomodulatory properties.

PTB vaccination has previously demonstrated neutrophil functionality enhancement, increasing phagocytosis and NET release (Ladero-Auñon et al., 2021). When PMNs bind to the bacteria and ingest them by phagocytosis, respiratory burst is started, and ROS are generated (Andrés et al., 2022). ROS fight infection by killing the microbe, mediating with inflammatory responses, activating cellular signaling, releasing proinflammatory cytokines, and activating NET formation (Li and Trush, 2016). In the present study, vaccination, in the absence of probiotics, was the only treatment able to induce an increase of ROS, and the effect was maintained for at least 3 months. In contrast, this effect was absent when the animals had been supplemented with *Dietzia*. Recent studies have shown that probiotics can exert antioxidant activities inhibiting ROS production (Wang et al., 2017), such as the case of a yeast inhibiting the release of ROS by human PMNs (Jensen et al., 2008) or the *Bacillus* SC06 strain that attenuated the production of ROS in rat jejunum and IEC-6 cells by MAPK-p38 activation (Wu et al., 2019). The results obtained in the present study for peripheral phagocytes indicate that *Dietzia* alone might have antioxidant properties and that it might be able to immunomodulate the effect of vaccination, inhibiting the production of ROS enhanced by vaccination. This can be beneficial in the sense that it can maintain host cell survival since low ROS levels activate cell survival pathways and high ROS levels activate cell death signaling pathways, but it can also be detrimental if this means losing bactericidal activity.

Actually, a similar effect can be observed regarding PMN bactericidal capacity against *S. aureus* as it was only increased in the CV group, meaning that both specific (ROS production against Map sonicate) and non-specific (killing against *S. aureus*) immune responses are being activated by PTB vaccination, and in both cases these responses are hampered when *Dietzia* is administered after vaccination. This ability to exert antimicrobial activity against other non-related pathogens is not surprising since Silirum^®^ was able to increase NETosis and phagocytosis of peripheral PMNs *ex vivo* after stimulation with Map and other non-related pathogens (Ladero-Auñon et al., 2021) and has proven to reduce non-specific mortality in cattle (Juste et al., 2021) as mentioned previously. These results are in agreement with studies involving Bacillus Calmette–Guérin (BCG), another mycobacterial vaccine that has shown protection against *S. aureus* (Covián et al., 2019).

Although the humoral response has not been considered to be an important part of protection against Map infection, some studies suggest the contrary (Juste and Perez, 2011; Pooley et al., 2019; Jolly et al., 2022). Humoral response has shown a positive association with the reduction of bacterial burden in both sheep and goats (Juste and Perez, 2011) and has been suggested to be essential for successful vaccine protection in sheep (Pooley et al., 2019; Links et al., 2021). Actually, the addition of antibodies obtained from Map-positive cows has shown to reduce Map invasion and consequent early histological changes in an ileal short-term loop model (Jolly et al., 2022). More specifically, the increase of mucosal immunity and antibody production could aid phagocytes in their fight against Map (Ladero-Auñon et al., 2021). In our study, both vaccinated groups presented the highest reactivity against Map-specific antigens and against PPA-3 1 month after vaccination and before challenge. This was expected for CV, as shown in previous studies (Arrazuria et al., 2016; Ladero-Auñon et al., 2021), but was unknown for CV-PR. Probiotic administration therefore seems to not affect lymphocyte B or plasma cells. Regarding the group treated only with probiotic (PR), the reactivity of IgG and IgA against Map antigens before infection did not increase.

Lactate has been observed to modulate immune responses in the microenvironment and systemically (Buck et al., 2017) and has been related with cell proliferation and metabolic demand (Heiden et al., 2009). Lactate has shown to alter human macrophage metabolism, improving their ability to kill *Mycobacterium tuberculosis* (Ó Maoldomhnaigh et al., 2021). In the present study, groups only vaccinated (CV) and vaccinated in combination with probiotic (CV-PR) showed an increase of lactate production at PT1 (1 month after treatment initiation), although significant differences were only detected for CV-PR. This increase in lactate is indicative of an increase of metabolic activity that can be originated by immune cell activation and increased granulopoyesis as that proven for BCG (Brook et al., 2020). The high levels of lactate in the CV-PR group that indicate a higher metabolic demand can be originated by blood cell types other than PMNs.

In any case, *in situ* findings in SR indicate that both treatments CV and CV-PR are beneficial for Map clearance as these animals present the lowest Map burden compared to the rest of the groups. The administration of the probiotic before challenge did not favor bacterial removal, and what is more, the detection of Map DNA and viable Map was highest in the PR group, indicating that *Dietzia* administered in these conditions is detrimental. The probiotic seems to protect Map, which may be due to the anti-inflammatory/antioxidant properties of the probiotic or to the occurrence of a shift in microbiota that benefits Map survival. CV was not fully effective probably because Map detection was performed at the early stages of the disease (3 months after challenge) and also because strain 832 is more virulent than Map K10, in contrast to the findings by Arrazuria et al. (2016), where Map detection was performed 6 months after challenge, and significant differences between the CV and CC group were observed. These findings support that CV modulates the immune response but does not produce sterile immunity.

Regarding macrophage polarization and proliferation, the different treatment regimens did not cause a clear differentiation 3 months after challenge due to the high individual variability and probably to the length of the experiment. The discrepancies between the present results and those presented in Arrazuria et al. (2020) can be due to the different sampled time points, indicating that between 3 and 6 months after challenge, there might be a switch in macrophage polarization from M2 to M1, which can be originated from the continuous release of IFNγ. Vaccination (CV) interfered with macrophage recruitment as seen by the calprotectin decrease, and the administration of the probiotic after vaccination reduced IFNγ production, indicating that the probiotic might inhibit the activation of proinflammatory responses of vaccination.

**Figure 8:**
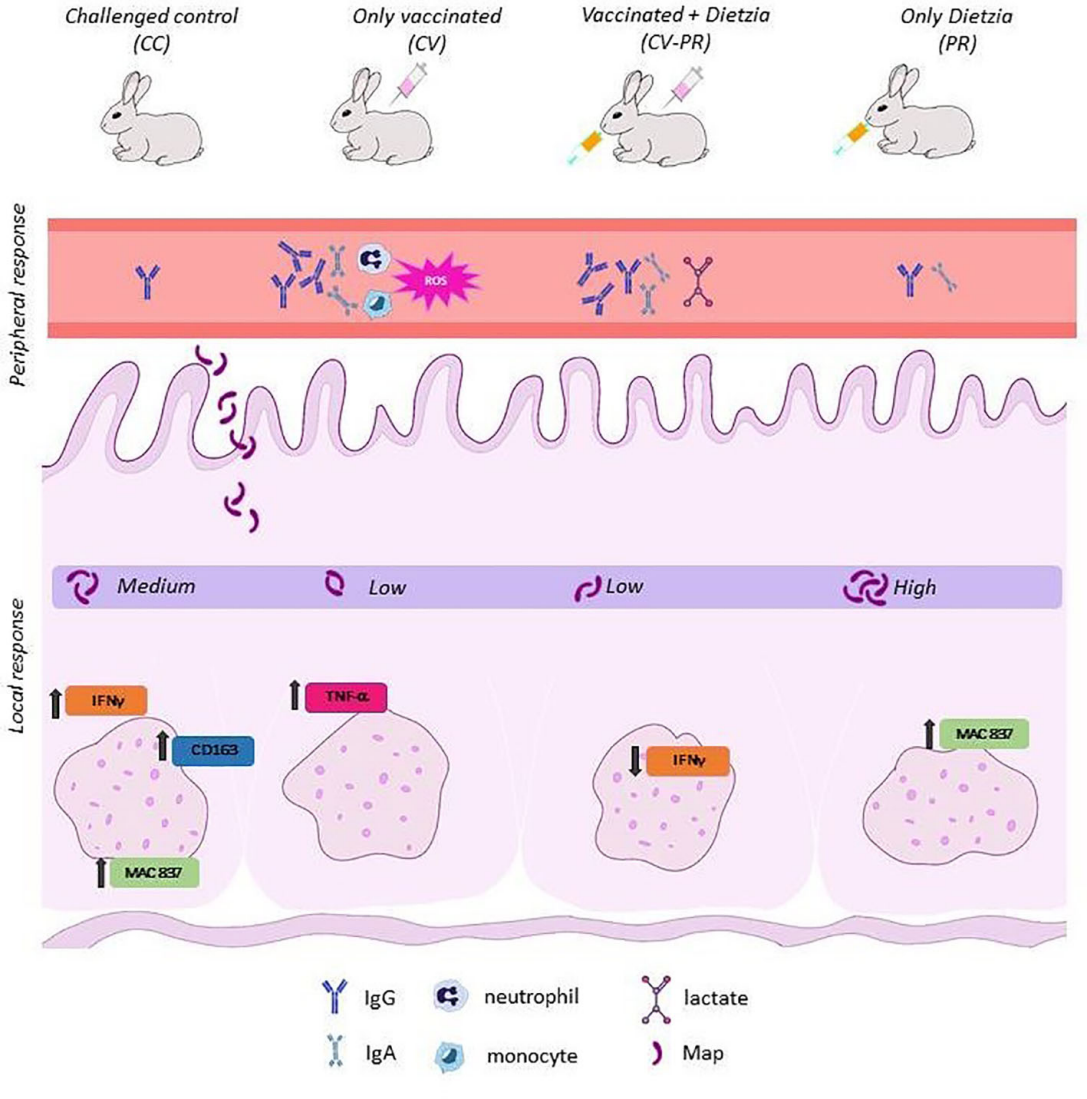
Summary findings for each experimental group.

The differences observed in peripheral and local responses between treated groups (**Figure 8**) might be due to the modulation of different immune response pathways. CV presented a low bacterial burden concomitant with a higher ROS and bactericidal activity in PMNs and the lowest calcoprotectin staining indicative of less active lesions. These findings suggest that PMN activity can be related to protection and may play an important role in the elimination of Map in the initial stages of infection. However, probiotic administration after vaccination (CV-PR) decreased phagocyte function compared to only vaccination, and this did not affect the protective properties of the vaccine as seen by the bacterial burden in tissues. Furthermore, the CV-PR group presented the highest lactate levels at PT1 and the lowest IFNγ levels in SR, findings that can be linked to macrophage polarization as seen in another study that shows that lactate is able to promote the transformation of activated macrophages to M2 phenotype and the repair of the intestinal mucosal barrier protecting intestinal tissue from inflammation (Zhou et al., 2022). It could be that, in the CV-PR group, the probiotic has shifted the immune response to other protective mechanisms that lead to a similar outcome as in the CV group. In any case, both groups share antibody production, and antibodies have been suggested to play a role in protection (Pooley et al., 2019; Links et al., 2021; Jolly et al., 2022).

Ultimately, the different effects observed in the studies performed by Click et al. (Click and Van Kampen, 2010; Click, 2011b, a) with *Dietzia* and those presented here can be due to differences in the experimental design (animal species, moment, timing and amount of probiotic administration). In any case, in these particular experimental settings, the PR group showed the worse outcome. Other authors have also demonstrated that the administration of a probiotic is not necessarily always beneficial for the host (Zawistowska-Rojek and Tyski, 2018), and the failure or benefit may depend on the particular strains (Stadlbauer, 2015). In conclusion, probiotics should always be assayed in the context of each particular disease (considering both host species and pathogen) since they are immunomodulators that can affect immune response pathways in different ways.

## Data availability statement

The raw data supporting the conclusions of this article will be made available by the authors, without undue reservation.

## Ethics statement

The animal study was approved by Ethics Committee at NEIKER (NEIKER-OEBA-2018-0001) Regional Council (BFA-38012). The study was conducted in accordance with the local legislation and institutional requirements.

## Author contributions

MO: Data curation, Formal Analysis, Investigation, Methodology, Writing – original draft, Writing – review & editing. EM: Data curation, Investigation, Methodology, Writing – review & editing. MM: Investigation, Methodology, Writing – review & editing. IL-A: Methodology, Writing – review & editing, Investigation. MFu: Methodology, Writing – review & editing, Investigation. MFe: Methodology, Writing – review & editing, Investigation. JB: Investigation, Methodology, Writing – review & editing, Formal Analysis. NE: Conceptualization, Data curation, Formal Analysis, Funding acquisition, Investigation, Methodology, Project administration, Supervision, Writing – original draft, Writing – review & editing.
