# Supplementary material for: Probiotic bacteria can modulate immune responses to paratuberculosis vaccination
Source: Front Cell Infect Microbiol. 2024 Jun 4;14:1394070. doi: 10.3389/fcimb.2024.1394070 (PMC11183331; doi:10.3389/fcimb.2024.1394070)
Supplement: Supplementary file 1 [file DataSheet_1.pdf]

Supplementary Material

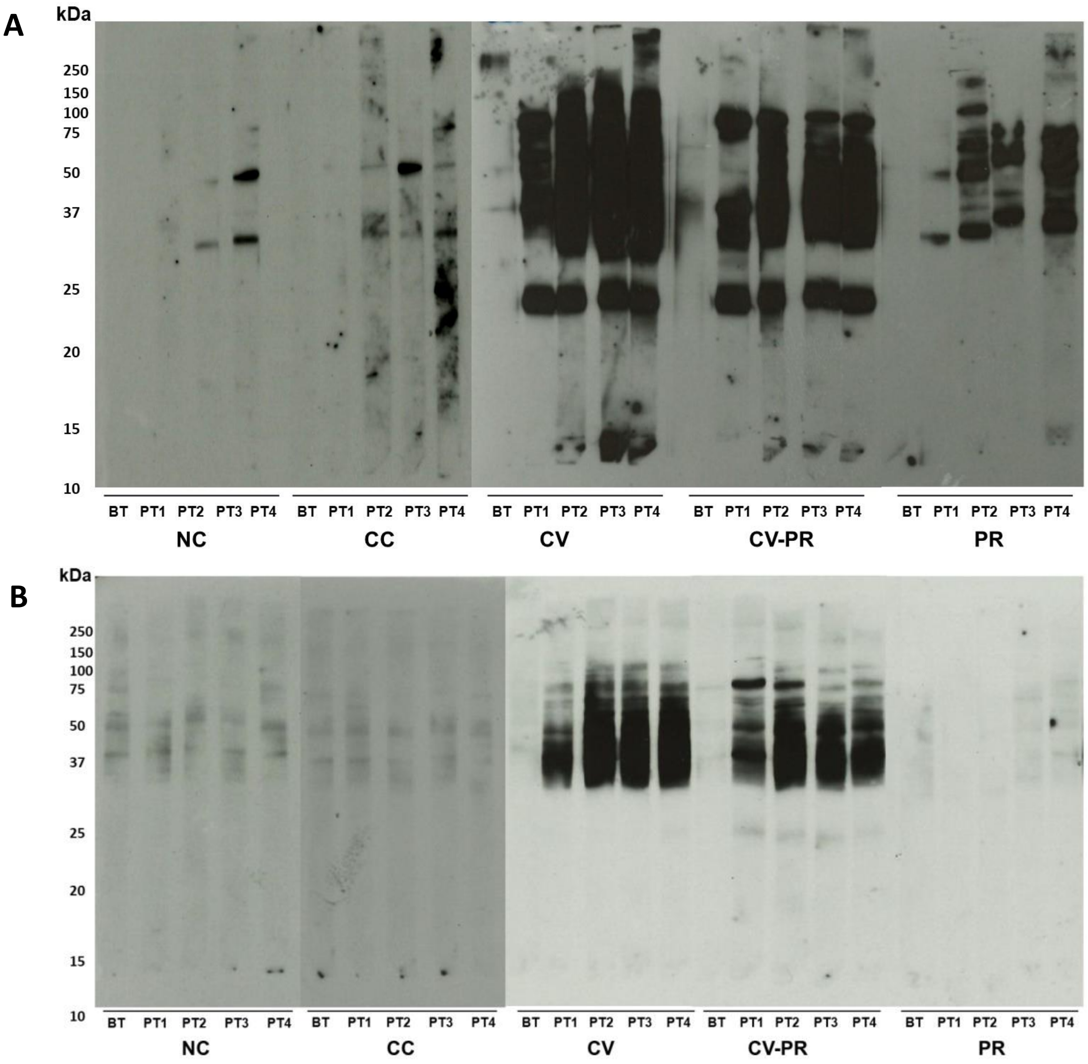

Supplementary Figure 1. Map antigen detection by immunoblot. (A). Anti-IgG reactivity. Strips of Map protein extract incubated with pools of serum of each experimental group at each time point, **(B)** Anti-IgA reactivity. Strips of Map protein extract incubated with pools of serum of each experimental group at each time point. Strips were incubated with serum pool dilution at 1:50; Recombinant protein G peroxidase at 1:800 and Goat Anti-Rabbit IgA alpha chain peroxidase at 1:5000.

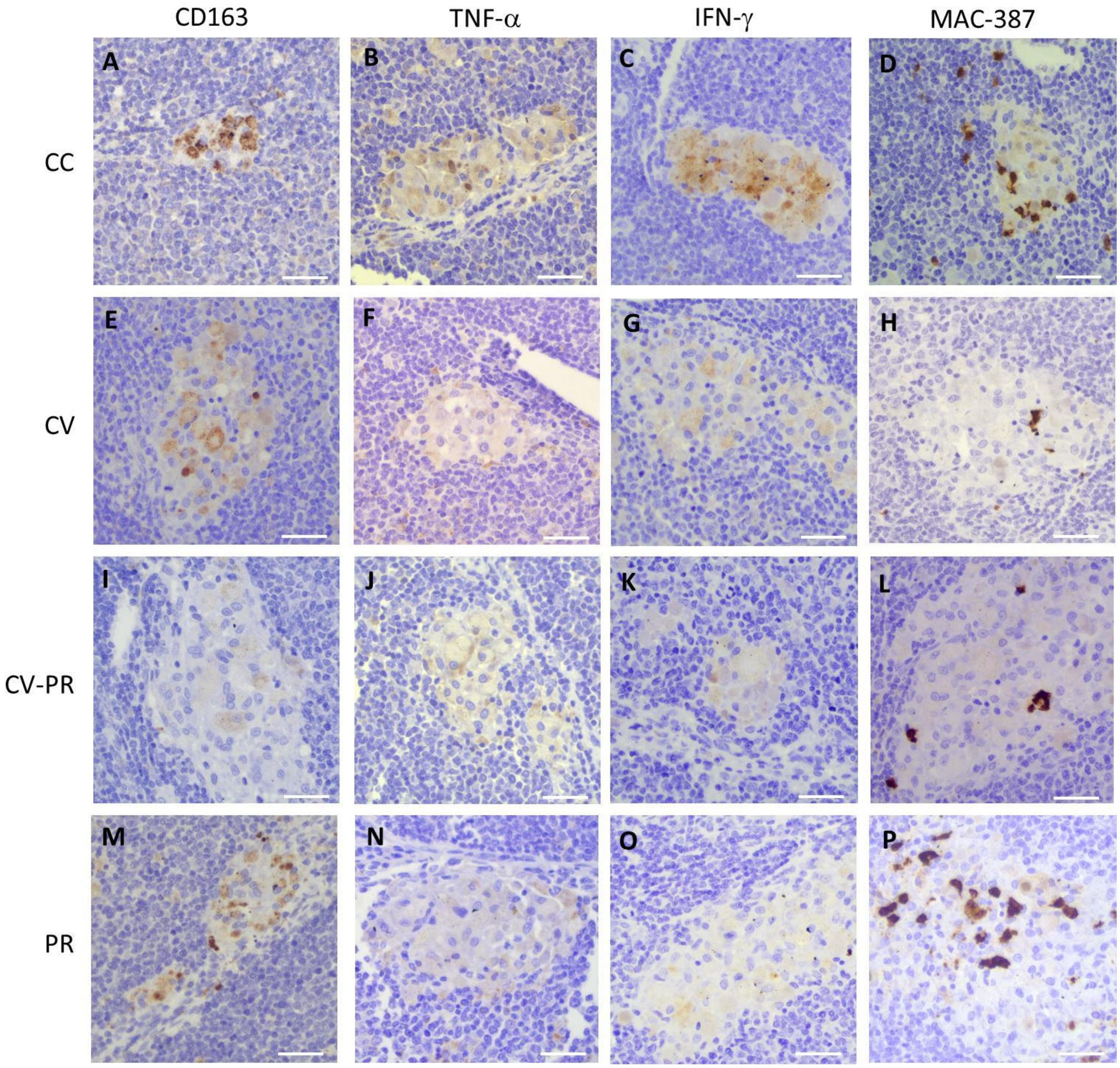

Supplementary Figure 2. Immunohistochemistry. Representative micrographs of the immunostaining in sacculus rotundus for each experimental group **(A-D)** challenged control; CC, **(E-H)** commercial vaccine; CV), **(I-L)** commercial vaccine in combination with probiotic; CV-PR and **(M-P)** treated with probiotic; PR and marker A, E, I and M are CD163; B, F, J and N are TNF-α; C, G, K and O are IFNγ and D, H, L and P are MAC387-calprotectin. (scale bar=50 μm).
